# The Oncogenic Potential of Human Cytomegalovirus and Breast Cancer

**DOI:** 10.3389/fonc.2014.00230

**Published:** 2014-08-25

**Authors:** Georges Herbein, Amit Kumar

**Affiliations:** ^1^Department of Virology and Department of Pathogens & Inflammation, UPRES EA4266, SFR FED 4234, CHRU Besançon, University of Franche-Comté, Besançon, France

**Keywords:** cytomegalovirus, breast cancer, macrophage, HCMV, inflammation

## Abstract

Breast cancer is the leading causes of cancer-related death among women. The vast majority of breast cancers are carcinomas that originate from cells lining the milk-forming ducts of the mammary gland. Numerous articles indicate that breast tumors exhibit diverse phenotypes depending on their distinct physiopathological signatures, clinical courses, and therapeutic possibilities. The human cytomegalovirus (HCMV) is a multifaceted highly host specific betaherpesvirus that is regarded as asymptomatic or mildly pathogenic virus in immunocompetent host. HCMV may cause serious *in utero* infections as well as acute and chronic complications in immunocompromised individual. The involvement of HCMV in late inflammatory complications underscores its possible role in inflammatory diseases and cancer. HCMV targets a variety of cell types *in vivo*, including macrophages, epithelial cells, endothelial cells, fibroblasts, stromal cells, neuronal cells, smooth muscle cells, and hepatocytes. HCMV can be detected in the milk after delivery and thereby HCMV could spread to adjacent mammary epithelial cells. HCMV also infects macrophages and induces an atypical M1/M2 phenotype, close to the tumor-associated macrophage phenotype, which is associated with the release of cytokines involved in cancer initiation or promotion and breast cancer of poor prognosis. HCMV antigens and DNA have been detected in tissue biopsies of breast cancers and elevation in serum HCMV IgG antibody levels has been reported to precede the development of breast cancer in some women. In this review, we will discuss the potential role of HCMV in the initiation and progression of breast cancer.

## Introduction

Breast cancer is the most frequent cause of cancer-related death among women. According to world health organization more than 5 million deaths have been attributed to breast cancer in 2011 (Global Health Estimates, WHO 2013). Breast cancer represents a heterogeneous disease. Based on the transcriptomic profile, breast tumors have been categorized into at least five intrinsic subtypes (1, 2). These subtypes include basal like, ERBB2+, normal breast-like, luminal subtype A, and B (1). Information regarding the intrinsic subtype of breast cancer in patients has prognostic significance and may help in designing personalized therapy in future (3). Familial history of breast cancer, hormonal replacement therapies, alcohol consumption, and radiation exposure are few risk factors associated with breast cancer (4). Biological entities especially viruses are also known to trigger various cancers in human. There are at least one dozen of viruses with established role in human malignancies [comprehensively reviewed in Ref. (5)]. Few viruses are suspected to play a role in the initiation or promotion of breast cancer (6). One of such viruses is human cytomegalovirus (HCMV).

Human cytomegalovirus (also called human herpesvirus 5) is a member of herpesviridae family (subfamily betaherpesvirinae). HCMV infects nearly 50–90% of the population worldwide. HCMV infection is either asymptomatic or causes mild discomfort. In certain situations where immunity is either immature or immunocompromised or suppressed, HCMV infection causes significant morbidity and mortality (7, 8). In the last decade, presence of HCMV genome and antigens has been reported in several kinds of human cancers. These human malignancies include breast cancer, brain cancer, prostate cancer, colon cancer, and salivary gland cancer (9, 10). In addition, we and others have shown the oncogenic transforming potential of HCMV *in vitro* (11–13). In this review, we will focus reader’s attention on the potential link between HCMV infection and the initiation and/or development of breast cancer.

## HCMV and Breast Cancer: A Volatile Relationship

Several attempts have been made to search for a virus responsible for breast cancer. In 1971, Moore and colleagues examined the milk samples from women with or without the history of breast cancer using electron microscopy. They found higher prevalence of virus like particles designated as “particle B” in the milk of women with familial history of breast cancer. Exact nature of “particle B” is still an enigma (14). With the rapid advancement in DNA and protein technology, presence of several viruses has been detected in breast cancer and normal tissue. The presence of human papilloma virus (HPV) (15, 16), Epstein–Barr virus (EBV) (17), human endogenous retroviruses (18), and more recently JC and BK human polyomaviruses (19) has been reported in patients derived breast cancer specimens (Table 1). However, contrasting findings are also available, which make the relation of viruses and breast cancer highly volatile.

**Table 1:** **A list of viruses that could potentially be involved in breast cancer**.

Virus	Reference
Epstein–Barr virus	(20, 21)
Human cytomegalovirus	(22, 23)
Human papillomavirus	(20, 24–26)
Simian virus 40	(27)
Human polyomavirus JC	(19)
Human polyomavirus BK	(19)
Human mammary tumor virus	(28, 29)
Merkel cell polyomavirus	(20)
Human endogenous retrovirus K	(18, 30)

Human cytomegalovirus has large protein repertoire that can initiate or promote neoplastic changes in a cell. Richardson hypothesized that incidence of breast cancer could be raised by late exposure to HCMV (31). This hypothesis was based on the incidence of breast cancer and its correlation with the seroprevalence of HCMV. Cox and colleagues investigated the correlation between levels of HCMV IgG with the development of breast cancer. They enrolled 399 women with invasive breast cancer and 399 controls. Results of their study suggest a statistically significant correlation with the elevation of HCMV IgG levels and development of breast cancer in some women (22) (Table 2).

**Table 2:** **HCMV prevalence in breast cancer patients**.

Study	Subjects	HCMV IgG positivity (%)	Mean IgG			HCMV DNA positivity (%)	Reference
			Sample I	Sample II^c^	Seroconversion	
1	Control women (*n* = 399)	82.5	1.18 OD^a^	1.22 OD^a^	*n* = 3	np	(22)
	Invasive breast cancer patients (*n* = 399)	78.7	1.09 OD^a^	1.19 OD^a^	*n* = 11	np	
2	Non-inflammation breast cancer (*n* = 49)	65	18.45 ± 15.7 IU/mL (*n* = 42)		–	53.1^b^	(46)
	Inflammation breast cancer (*n* = 28)	82	25.96 ± 24.50 IU/mL (*n* = 28)		–	78.6^b^	

*^a^Positive control kit mean OD 0.825 ± 0.110*.

*^b^Starting material was cancerous tissue*.

*^c^Average time between two samples was 8.5 years in Ref (22)*.

*n, number of patients, np, not performed*.

Breast milk is the predominant source of HCMV transmission in human. Presence of HCMV has been detected in more than 90% of milk samples derived from women seropositive for HCMV (32, 33). Using polymerase chain reaction and Southern analysis, presence of HCMV DNA was reported in normal breast tissue (34). Data from Cobb’s laboratory provide a more direct evidence of breast epithelium as an important reservoir for HCMV in humans. They detected the presence of HCMV antigens (specifically in glandular epithelium) in surgical biopsies of normal breast, breast with tumor, and normal breast tissue from the breast cancer patients using immunohistochemistry. Prevalence of HCMV antigens was relatively higher in neoplastic epithelium of patients with breast cancer (23).

A recent report by Soderberg-Naucler laboratory demonstrated the presence of HCMV proteins and DNA in breast cancer and sentinel lymph node metastases tissue using immunohistochemistry and PCR. In their study, presence of HCMV antigens was restricted to metastatic tumor cells that are not consistent with previous reports (23, 35).

There are several reports where no correlation has been found between HCMV and breast cancer. For example, Antonsson and coworkers screened for the presence of several viruses (including HCMV and EBV) in 54 fresh breast tissue samples using real time PCR. They did not detect the presence of HCMV in any sample (20). In another instance, Utrera-Barillas and colleagues investigated the association of presence of HCMV DNA with breast cancer progression in primary breast cancer biopsies using real time PCR (36). They detected HCMV DNA in only 2 cases out of 27 breast cancer specimens. They found no significant correlation between HCMV presence and breast cancer progression. Variation in tissue handling, sample size, PCR primer designing, and sites of tissue sampling could be responsible for the discrepancy in results among different laboratories (37).

Collectively, data from *in vitro* and *in vivo* suggest that HCMV may be involved in the initiation or progression of breast cancer. However, in order to obtained conclusive results, clinical findings need to be analyzed on large cohorts and *in vitro* findings need further validation in animal models.

## HCMV Cell Tropism and Breast Cancer

**Figure 1:**
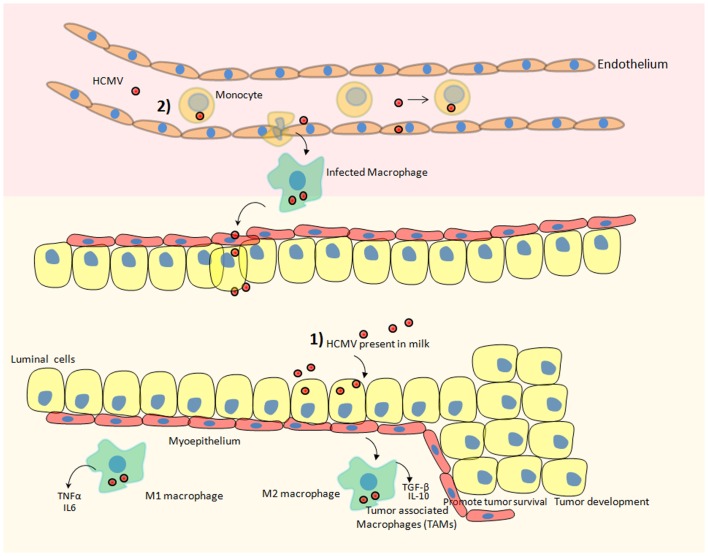
**Hypothetical scheme of HCMV dispersal and its possible involvement in the development of breast cancer**. Two potential scenarios could ultimately result in breast tumor after HCMV infection. (1) HCMV that is present in the milk could directly infect the mammary epithelial cells lining the duct responsible for converting most precursors into milk constituents and transporting them to the mammary lumen. Subsequently, macrophages present in breast tissue could be also infected by HCMV favoring a protumoral microenvironment. (2) HCMV present in blood (viremia) could infect circulating monocytes. Upon migration of infected monocytes into breast tissue, HCMV-infected macrophages could transmit the virus to mammary epithelial cells. Additionally, monocytes/macrophages are regarded as a prominent reservoir of HCMV infection. HCMV infection of monocytes/macrophages can reprogram them to acquire M1/M2 characteristics. M1 macrophages secrete pro-inflammatory cytokines and M2 macrophages secrete immuno-suppressive factors that can promote the progression of breast cancer. Tumor-associated macrophages (TAM) that are of poor prognosis during breast cancer and fuel the progression of the disease could be preferentially activated by HCMV.

Human cytomegalovirus is known to infect virtually most organs of the human body including blood, brain, breast, colon, eye, kidney, liver, and lung. Therefore, HCMV exhibits broader tropism. Several reports indicate the replication of HCMV in various cells ranging from monocytes to neural stem cells (23, 38–40). Monocytes and macrophages are widely recognized as important HCMV reservoirs responsible for the dissemination of the virus throughout of the body (41, 42). Furthermore, infection of monocytes by HCMV has potential to reprogram monocytes, resulting in polarization toward inflammatory macrophages (M1) that also exhibits properties of immunosuppressive macrophage (M2) (43) (Figure 1). This polarization is mediated by induction of NF-κB and PI3K activities in monocytes upon HCMV infection (43). M1 macrophages secrete inflammatory factors including TNFalpha, IL-6, and nitric oxide synthase 2 (Figure 1). Prolonged secretion of these cytokines is often linked with the development of cancer [reviewed in Ref. (44)]. We have also observed the positive correlation among the seroprevalence of HCMV IgG, elevated IL-6 levels, and incidence of liver cancer in a patient oriented study (45). In another instance, El-Shinawi and co workers investigated the prevalence of HCMV infection in patients with inflammatory breast cancer (IBC) and non-IBC invasive ductal carcinoma (IDC) patients. They observed the higher prevalence of HCMV IgG in patients with IBC than IDC patients (Table 2). Furthermore, they detected higher levels of DNA and activation of NF-κB in cancerous tissue isolated from IBC as compared to IDC patients. Increased activation of NF-κB can be a result of HCMV infection of breast cells or indirectly by cytokine production in the tumor microenvironment (46).

Macrophages associated with the tumorous environment are known as tumor-associated macrophages (TAM). The predominant fractions of TAMs are M2 polarized macrophages. Upon infiltrating tumor surroundings of breast cancer cells, macrophages may acquire M2 state. These M2 macrophages secrete high levels of immunosuppressive cytokines, e.g., IL-10, TGF-beta, and little amount of pro-inflammatory cytokines [reviewed in Ref. (47)] (Figure 1). These M2 macrophages being immunosuppressive in nature indirectly favors the development of breast cancer. Since HCMV can infect normal breast and malignant breast tissue therefore the role of HCMV in favoring TAMs phenotype (by inducing M2 polarization) and breast cancer progression is highly speculated. For instance, the clinical isolate HCMV-DB displays preferential macrophage tropism, triggers M2 activation state, and stimulates the upregulation of the proto-oncogene Bcl-3 (38).

Indeed, TAMs are considered as an important therapeutic target in breast cancer. Luo and colleagues have identified legumain a stressed molecule overexpressed by TAMs. In addition, the application of DNA vaccine against legumain has been shown to dramatically reduce tumor angiogenesis in animal model (48).

Taken together, these primarily data suggest that the potential route of viral dispersal to breast tissue and involvement of HCMV in breast cancer progression.

## Potential Oncogenes in HCMV Genome

**Figure 2:**
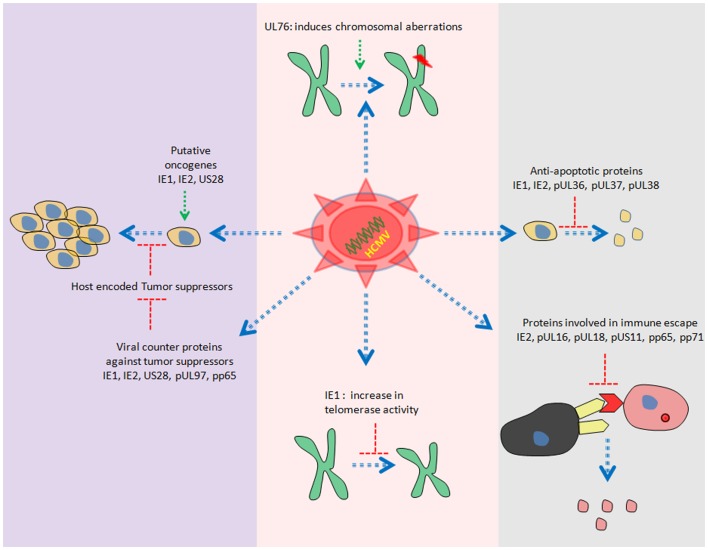
**Overview of diverse HCMV proteome involved in modulation of host cell controlled growth**.

Human cytomegalovirus is a double stranded DNA virus with ~240 kb of genomic information. Recent study reveals the presence of more than 700 translated ORFs (49) in HCMV genome, which is more than double of previous predictions (50, 51). HCMV gene products have been reported to be involved in cell cycle dysfunction, genome instability, cell immortalization, inhibition of important cellular players involved in apoptosis and immune invasion (52–55) (Figure 2).

Several members of HCMV proteome have oncogenic properties. For instance, stable expression of US28 in NIH3T3 cells has been shown to induce transformed phenotypes. In addition, injection of NIH3T3 cells stably expressing US28 in mice leads to tumor formation (56). One of the responsible mechanisms for induction of tumor growth could be the activation of IL6–JAK1–signal transducer and activator of transcription 3 (STAT3) axis by US28 *in vitro* and *in vivo* (57). Furthermore, we have also observed similar findings in primary human hepatocytes and HepG2 cells upon HCMV infection (13).

Other gene products with oncogenic potential are immediate early (IE) 1 and IE2. Shen and colleagues have observed the transformation of primary baby rat kidney (BRK) cells upon transient expression of IE1, IE2, and adenovirus E1A proteins. Interestingly, they were not able to detect the presence of IE1/IE2 DNA in clonal cell lines derived from transformed BRK foci. The unconventional “hit and run” mechanism has been proposed to explain the transformation by IE1/IE2 (58). In addition, ectopic expression of IE1 induces telomerase mRNA and enhance telomerase activation in normal human diploid fibroblasts *in vitro* (59) (Figure 2).

Purified HCMV virions have reported to induce chromosomal breaks in primary human foreskin fibroblasts (HFF) (60). Data from Spector laboratory further revealed that only viral entry but not viral gene expression was prerequisite to induce chromosomal breaks in HFF (60). UL76 (one of the virion associated proteins) stable expression in human glioblastoma cells has been shown to induce chromosomal breaks (61) (Figure 2).

Taken together, data suggest that the presence of several HCMV proteins that have ability to induce transformation. Of note, transient or stable expression of a particular viral gene may not truly represent the natural HCMV infection scenario. Rigorous experimentations are needed to elucidate the exact function of these viral proteins in tumor tissue.

## Antiviral Drugs Against HCMV as Novel Breast Cancer Therapies

Presence of HCMV in various cancerous tissues raises the possibility of using anti-HCMV drugs in targeting cancer cells. In breast cancer and HCMV infection, there are several common pathways that are activated. For example, aberrant activity of STAT3, PI3K, NF-κB, MAPK, and Wnt driven cascade is observed in both HCMV infection and breast cancer (Table 3). Therefore, drugs that target these pathways should have significant impact on both HCMV infection and tumor progression. There are several compounds at preclinical or clinical trial stage that show significant impact on cancer development (Table 3). One of such FDA approved drug is sorafenib, a multi kinase inhibitor that affects several signaling cascades. Sorafenib is known to inhibit the replication of HCMV in several cell types *in vitro* (62). In addition, sorafenib inhibits cell death and induce apoptosis in several breast cancer cell lines including MCF-7 and MDA-MB-231 (63). In a phase II clinical trial involving patients with HER2 negative breast cancer, combination of sorafenib and cepacitabine has been reported to improved progression free survival in patients (64). Furthermore, phase III clinical trial (Clinicaltrials.gov, NCT01234337) with reduced dose of sorafenib has been also started (65).

**Table 3:** **Major signaling pathways targeted by HCMV and activated in breast cancer**.

Signaling pathways altered upon HCMV infection	Effector viral protein	Drugs/inhibitors targeting signaling pathways in breast cancer	Reference
JAK-STAT3	US28, IE1	Sorafenib	(57, 66, 67)
		FLLL31^a^, FLLL32^a^, BP-1-102^a^	(68, 69)
PI3K-AKT	IE1, IE2	Buparlisib (BKM120)^b^	(70, 71)
		NVP-BEZ235^a^	(72, 73)
		MK-2206^b^	(74)
MAPK-ERK	gB	Sorafenib	(75–79)
		CI-1040 (PD184352)^b^	(80)
Wnt/beta-catenin	Not known	XAV939^a^	(81, 82)

*^a^Preclinical stage*.

*^b^In clinical trial*.

Signal transducer and activator of transcription 3 is an important transcription factor that governs genes responsible for cell cycle progression and apoptosis. Upregulation of pSTAT3 has been reported in several kinds of malignancies including breast cancer (83). In addition, activation of IL6–JAK–STAT3 axis has been observed upon HCMV infection in several cell types (13, 57). Involvement of viral proteins IE1 and US28 in modulating this signaling axis has been suggested (57, 66) (Table 3). Moreover, recent work demonstrates the activation of STAT3 in a triple negative breast cancer cell line (MDA-MB-231) upon treatment of cmvIL-10 (84). cmvIL-10 is encoded by ORF UL11a and is homolog to human IL-10 (85). Furthermore, exposure of cmvIL-10 to these breast cancer cell lines resulted in increase in cell proliferation and decrease in apoptosis.

Lin and colleagues designed two inhibitors designated as “FLLL31” and “FLLL32” derived from curcumin. These inhibitors bind specifically to Janus kinase 2 and STAT3 Src homology-2 domain that are indispensable for STAT3 dimerization and downstream signal transduction (68). *In vitro* application of these inhibitors has shown to inhibit STAT3 activation, cell invasion, and colony formation in breast cancer cell lines (68). Even results were promising in animal models. Same group has recently developed another STAT3 inhibitor termed BP-1-102 that represses the tumor growth of breast cancer cells in xenografts (69).

Another important signaling axis pertaining to homeostasis is PI3K–Akt axis. PI3K driven signaling cascades play an important role in cell growth, differentiation, glucose metabolism, and chemotaxis (86). In breast cancer, aberrant expression of PI3K and its downstream signaling partners is frequently observed. Involvement of IE1 and IE2 in activation of PI3K pathway has been also suggested (70). Several PI3K inhibitors are being tested against various cancers including breast cancer in clinical trials (Table 3). However, impact of these inhibitors in HCMV replication is not assessed.

In addition to above mentioned signaling pathways, impact of HCMV infection on Wnt pathway has been investigated (81, 87). HCMV-infected fibroblasts and human placental extravillous trophoblasts (81) exhibited decreased levels of Wnt 5a/b, Wnt driven beta-catenin, and total as well as phosphorylated form of lipoprotein receptor related protein 6 (87). In addition, treatment of cells with Wnt modulators (monensin, nigericin, and salinomycin) resulted in inhibition of HCMV replication in fibroblasts, suggesting important role of Wnt signaling partners in HCMV replication. Wnt plays important role in cell growth, cell fate determination, and tumorigenesis. Upregulation of Wnt pathway genes have been observed in breast cancer cell lines and patient samples (88). Collectively, this makes Wnt pathways an attractive tool in targeting HCMV and breast cancerous cells simultaneously. For example, XAV939 a small molecule tankyrase inhibitor has been shown to attenuate Wnt pathways in several breast cancer cell lines (82). However, effect of XAV939 in HCMV replication is not known yet and needs further investigations. Development of resistance against a particular drug during cancer progression is frequent. Therefore, there is need to formulate feasible combinatorial therapies against breast cancer.

## Conclusion

Several clinical and experimental investigations suggest the involvement of HCMV in various malignancies including breast cancer. *In vitro* and animal models suggest the presence of potent oncogenes genes in HCMV genome. Significance of higher prevalence of HCMV in breast cancer tissue is poorly understood. Whether it is just an “epiphenomenon” or crucial player in the progression of cancer needs further investigations.

## Author Contributions

Georges Herbein and Amit Kumar wrote the manuscript. Both authors read and approved this manuscript.

## Conflict of Interest Statement

The authors declare that the research was conducted in the absence of any commercial or financial relationships that could be construed as a potential conflict of interest.
